# Serine phosphorylation of the RhoGEF Trio stabilizes endothelial cell-cell junctions

**DOI:** 10.1080/21541248.2023.2242166

**Published:** 2023-08-01

**Authors:** Anna E. Daniel, Werner J. van der Meer, Lynn Wester, Vivian de Waard, Maartje van den Biggelaar, Jaap D. van Buul

**Affiliations:** a Department of Molecular Hematology, Sanquin Research and Landsteiner Laboratory, Academic Medical Centre, University of Amsterdam, Amsterdam, The Netherlands; b Department of Medical Biochemistry, Amsterdam UMC, University of Amsterdam, Amsterdam, The Netherlands; c Section Molecular Cytology at Swammerdam Institute for Life Sciences, Leeuwenhoek Centre for Advanced Microscopy, University of Amsterdam, Amsterdam, The Netherlands

**Keywords:** Trio, Rac1, serine, phosphorylation, endothelium

## Abstract

The RhoGEF Trio is a large multi-domain protein and an activator of the small GTPases Rac1, RhoG, and RhoA. Although Trio has been implicated in many cellular mechanisms like leukocyte transendothelial migration, cell-cell junction stability, lamellipodia formation, axon outgrowth, and muscle fusion, it remains unclear how Trio is activated. Using stable isotope labelling by amino acids in cell culture (SILAC)-based mass spectrometry analysis of endothelial cells, we identified two serine residues (S1785/S1786) located in between the two exchange domains of Trio that were highly phosphorylated upon short thrombin treatment. Using phosphomimetic Trio S1785D/S1786D double mutants, we did not find an increase in Rac1/RhoG activity, indicating that the phosphorylation events do not increase Trio exchange activity. However, we found that the Trio mutants localized more strongly at cell-cell junctions and prevented junction destabilization upon thrombin treatment, judged by junction linearity. Our data suggest that serine phosphorylation of Trio potentiates the localization of Trio to junctional regions, resulting in locally promoting the exchange for Rac1 at junction regions and increasing endothelial cell-cell junction stability upon permeability-inducing reagents such as thrombin.

## Introduction

The Rho family of GTPases is a family of small (~21 kDa) signalling G proteins, and are involved in many processes which are relevant to cell migration, vesicle trafficking, transcription, protein secretion, cell-substrate adhesion and cell-cell adhesion [1]. Small GTPases are active when they are bound to GTP and possess the intrinsic catalytic activity to hydrolyse GTP to GDP. GTPase activating proteins (GAPs) accelerate this process [2]. Guanine nucleotide exchange factors (GEFs) activate small GTPases by promoting the exchange of GDP for GTP which renders GTPases capable of interacting with downstream effector proteins [3].

RhoGEFs are a growing family of approximately 70 proteins, outnumbering the 22 known Rho-GTPases [3,4]. GEFs have a common domain of about 200 amino acids termed the Dbl-Homology (DH) domain. The DH-domain enables these proteins to exert their nucleotide exchange activity [3,4]. The pleckstrin homology (PH) domain present in GEFs, involved in membrane lipid binding, can assist the exchange reaction and, in some cases, binds the GTPase itself. Furthermore, this domain binds other proteins as well as phospholipids. Some GEFs also possess an SH3 domain of approximately 50–70 amino acids and this domain is assumed to play a role in protein-protein interactions and in directing protein compartmentalization within the cell [5]. Most GEFs also possess protein-protein interaction domains next to the SH3 domain including SH2, and DOCK domains, which can result in direct compartmentalization and sub-cellular localization and thus function [5].

We recently showed that the RhoGEF Trio plays an important role in leukocyte transendothelial migration, controls the expression of inflammatory molecules on the endothelium and regulates the dynamics of lamellipodia [6–8]. Trio was identified as a binding partner of the transmembrane tyrosine phosphatase LAR [9]. Due to its three putative catalytic domains, it was named Trio. Trio is a large protein of 350 kD which encompasses two Dbl-homology-Pleckstrin-homology (DH-PH) GEF units with different specificities. The N-terminal DH-PH unit (GEF1) mediates GDP for GTP exchange on Rac1 and RhoG, whereas the C-terminal DH-PH unit (GEF2) activates RhoA [10,11]. We have identified Trio as a crucial regulator for endothelial cell-cell junction stability [11]. Trio-deficient cells were unable to form novel cell-cell junctions after treatment with thrombin. Using electrical-substrate impedance sensing (ECIS), we showed that the recovery of endothelial monolayer resistance after thrombin stimulation was delayed in Trio-deficient endothelial cells. However, it remains unclear how Trio itself becomes activated.

Using high-resolution mass spectrometry, combined with stable isotope amino acids in cell culture (SILAC) and affinity-based phosphopeptide enrichment, we analysed potential serine phosphorylation of Trio after thrombin treatment. Interestingly, we found that serine residues 1785 and 1786 were highly phosphorylated after short-term thrombin treatment. Using specific phosphomimetic Trio mutants, we found that these residues were important not for Trio exchange activity but rather for localization to cell-cell junction regions to control endothelial cell junction stabilization.

## Results

**Figure 1.**
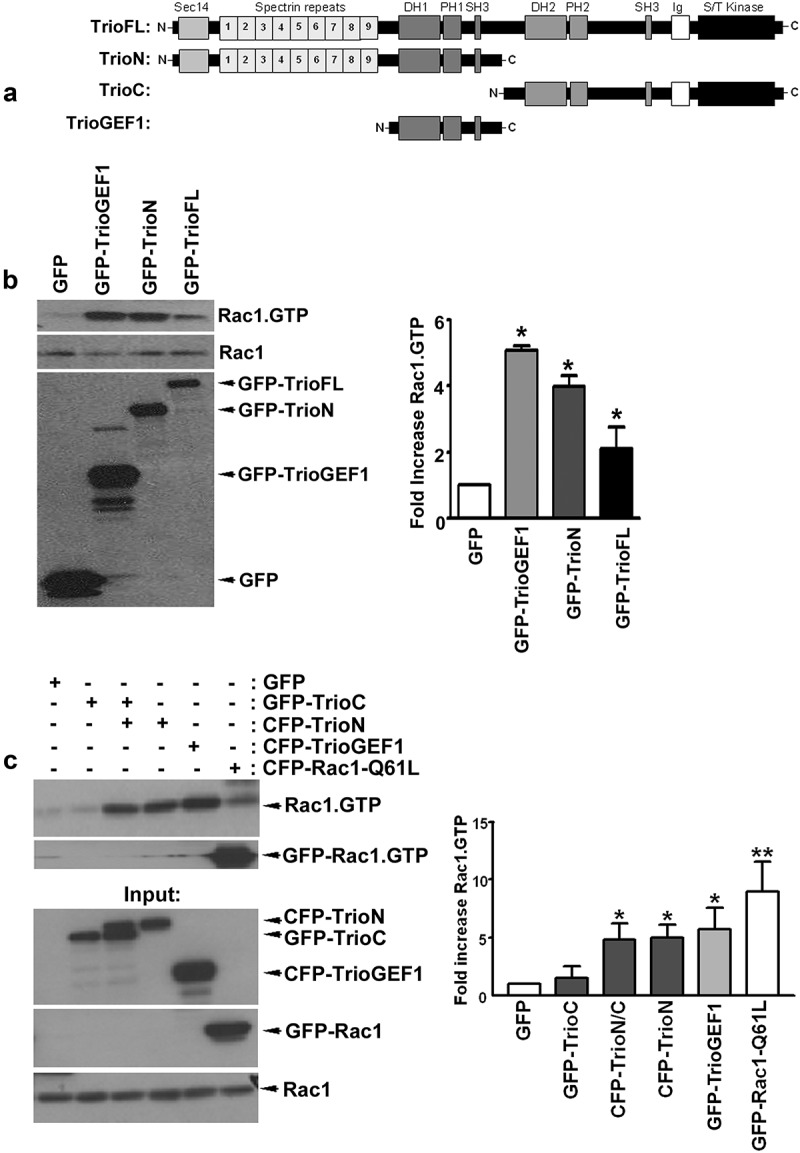
Trio activates endogenous Rac1 (**a**) Schematic overview of the TrioFL protein. TrioN encodes the N-terminal region, including the GEF1 domain. TrioC represents the C-terminus of Trio including the GEF2 domain and TrioGEF1 represents the GEF1 domain including an SH3 domain. (**b**) HEK293T cells were transfected with GFP-TrioGEF1, TrioN or TrioFL as indicated and active Rac1 (Rac1.Gtp) levels were determined. Western blot analysis showed that all Trio constructs activate endogenous Rac1. (**c**) HEK293T cells were transfected as indicated and active Rac1 levels were determined. Western blot analysis showed that TrioN activates endogenous Rac1 independently of TrioC. Input: Upper panel shows expression of Trio constructs. Lower panels show expression of Rac1 in total cell lysates. (b) and (c) Quantification of endogenous Rac1 levels; the values were normalized to the active Rac1 level in HEK293T cells transfected with GFP. Data are mean + SEM of three independent experiments. *P<.05; **p < 0.01.

To explore how RhoGEF Trio activity is regulated, we constructed several truncation mutants (Figure 1A). Using these mutants including the wt variant, we measured the ability of Trio to activate Rac1, one of its well-known downstream effectors. Expression of the Trio constructs containing the GEF1 domain, i.e., TrioFL (Trio-full length is wild type Trio), TrioN (the N-terminal part of Trio) and TrioGEF1 domain only, promoted the GTP loading of endogenous Rac1 (Figure 1B). Co-transfection of the GEF1 domain together with TrioN did not additionally increase Rac1-GTP levels, indicating that the loading of GTP on Rac1 is most likely limiting here (Figure S1A). To study potential auto-inhibitory effects of the second GEF domain on the first GEF domain, we co-expressed the C-terminus of Trio (TrioC) together with TrioN in HEK293T cells and measured endogenous Rac1 activity. No change in the ability of TrioN to exchange GDP for GTP on Rac1 was measured in the presence or absence of TrioC, indicating that the presence of the C-terminus of Trio did not directly inhibit the activity of the GEF1 domain (Figure 1C).

**Figure 2.**
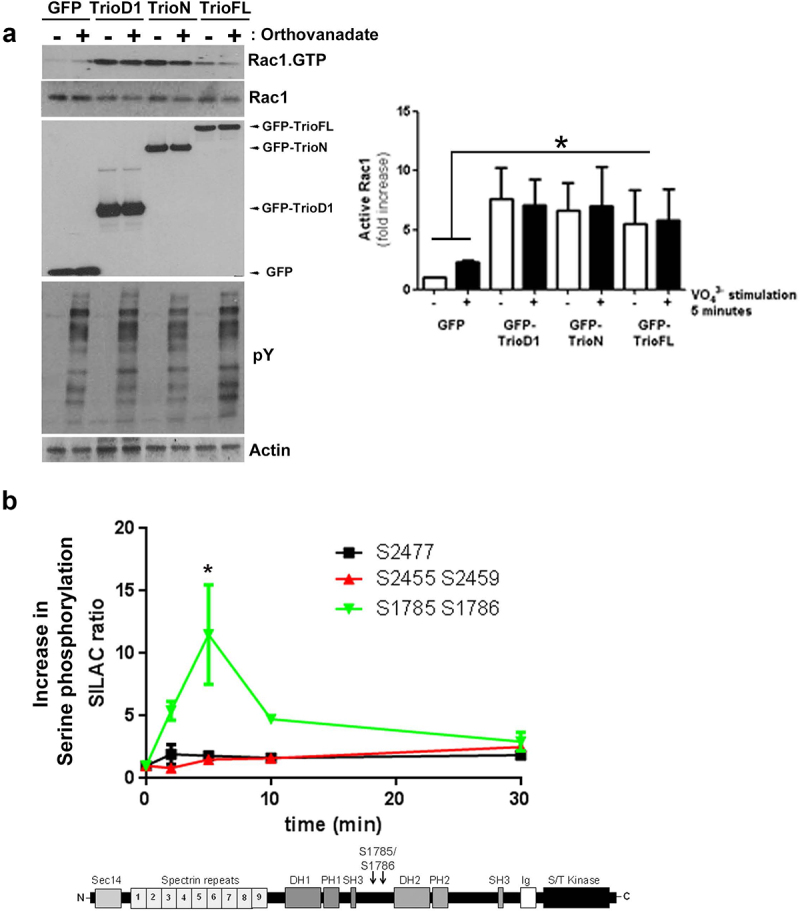
Pervanadate does not trigger Rac1 activation. (**a**) HEK293T cells were transfected and treated with pervanadate as indicated. No increase in Rac1.GTP loading is detected for any of the Trio constructs after pervanadate treatment. Lower panels show input and pY staining shows efficient pervanadate treatment. Actin was used as a loading control. Quantification of endogenous Rac1 levels; the values were normalized to the active Rac1 level in HEK293T cells transfected with GFP. Data are mean + SEM of three independent experiments. *P<.05. (**b**) SILAC-based mass spectrometry analysis of thrombin-treated HUVEC showed a significant increase (*p < 0.01) in serine phosphorylation on Trio residues S1785 and S1786, compared to serine residues (S2455 and S2459 or S2477) that remained unphosphorylated. The y-axis indicates fold increase in serine phosphorylation and drawing of Trio FL shows location of the 2 serine residues, in between the two exchange domains. Schematic drawing shows the location of the two serine residues in between the two GEF domains.

DeGeer and colleagues reported that tyrosine phosphorylation of Trio regulates netrin-1/DCC-mediated cortical axon outgrowth in N1E–115 neuroblastoma cells [10]. They showed that Trio was predominantly phosphorylated at tyrosine residue 2622 by the Src kinase Fyn [10]. We tested if direct Trio-induced Rac1 activation depends on tyrosine phosphorylation of Trio itself. For this, we overexpressed Trio mutants and treated the cells with the tyrosine phosphatase inhibitor pervanadate. Although tyrosine phosphorylation levels were increased in the cells treated with pervanadate, no increase in TrioFL-induced Rac1 activity was detected (Figure 2A). This is in line with data by DeGeer and colleagues who showed that the phospho-null Trio mutant (Y2622F) retained GEF activity towards Rac1, whereas this mutant did impair netrin-1-induced Rac1 activation [10].

Trio has many other sites that can be phosphorylated: apart from 61 tyrosine residues, there are 131 threonine and 230 serine residues in Trio. As our recent results showed that thrombin-induced recovery of endothelial cell-cell junctions was dependent on Trio-induced Rac1 activity [11], we focused on thrombin-induced phosphorylation of Trio in endothelial cells. In a previous study, we used stable isotope labelling with amino acids in cell culture (SILAC) and analysed the phosphorylation dynamics of the proteome downstream of thrombin stimulation in endothelial cells [12]. For Trio, we found five serine residues that showed changes in their phosphorylation pattern in response to thrombin treatment (i.e., S1785, S1786, S2455, S2459 and S2477; Figure 2B). A particularly high level of phosphorylation on serine residues S1785/S1786 was detected after five minutes of thrombin treatment (Figure 2B).

**Figure 3.**
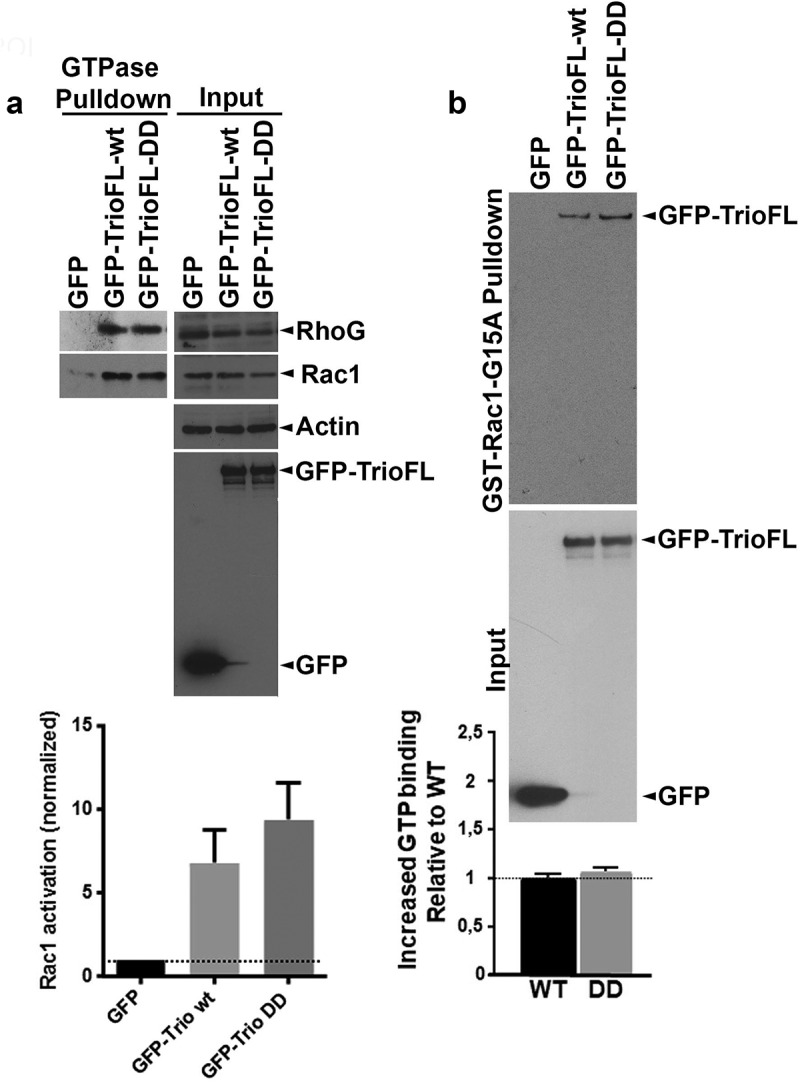
Serine phosphorylated Trio induces linear junctions. (**a**) Western blot analysis showed reduced Rac1.GTP and RhoG.GTP levels in HEK293 cells that were transfected with the phosphomimetic Trio mutant (GFP-TrioFL-DD). Quantification graph shows no significant increase in Rac1.GTP levels between WT and DD mutant. Experiment is carried out 5 times. *p < 0.01. (**b**) GST-Rac1-G15A (nucleotide-free Rac1 mutant) pulldown shows increased binding of the phosphomimetic mutant of Trio, compared to the Trio-WT. Input shows loading control. Experiment is done twice.

To analyse if the introduction of the point mutations at the sites of the serine residues enhances the activity of Trio directly, we measured the GTP-loading on Rac1, RhoG and RhoA after overexpressing the Trio-WT and mutant in HEK293 cells. Western blot analysis showed that the WT and DD mutant both promoted endogenous Rac1.GTP and RhoG.GTP levels (Figure 3A). However, no clear difference in GTP loading on Rac1 or RhoG was detected between Trio-WT and DD mutant. Next, we tested if the Trio-DD mutant may have increased intrinsic activity. For this we used a nucleotide-free GST-Rac1-G15A pull down assay. This assay is based on the fact that activated GEFs have the highest affinity for GTPases when they are in a nucleotide-free state. Therefore nucleotide-free GTPase mutants are used to pull down activated GEFs [13,14]. In line with the previous GTP assay, we found no difference in binding capacity between Trio WT and DD mutant to Rac15A (Figure 3B), indicating that the intrinsic activity of Trio was not altered by the DD mutant.

**Figure 4.**
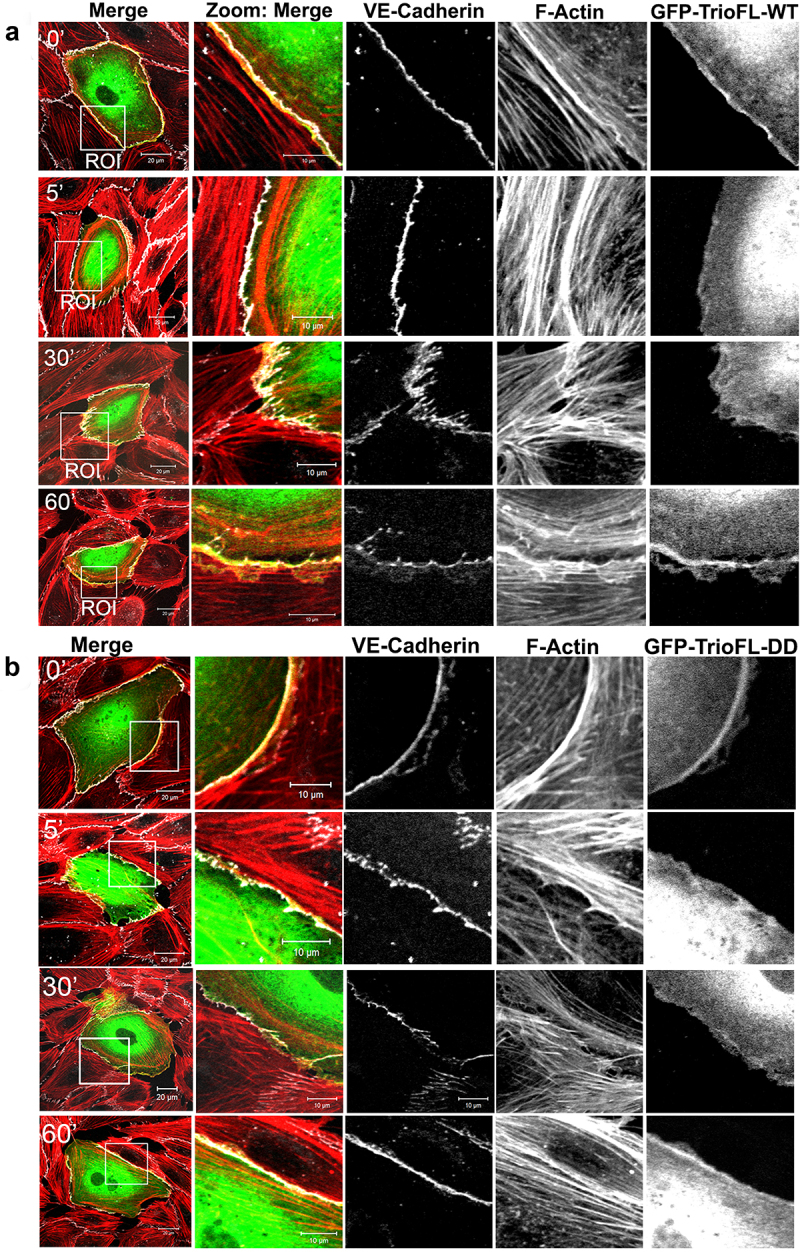
Phosphomimetic TrioFL-DD mutant reduces thrombin-induced Focal Adherens Junctions. HUVECs were transfected with GFP-tagged TrioFL-WT (**a**) or TrioFL-DD (phosphomimetic) mutant (**b**) as indicated. Cells were stimulated with thrombin (1 U/mL) for 5, 30 or 60 minutes or left unstimulated and then fixed, permeabilized and stained for VE-cadherin (white) and F-actin in red. ROIs (Regions of interest) show detailed phenotype of endothelial cell-cell junctions. Jagged phenotype corresponds to FAJ (focal adherence junction) phenotype. Scale bars are 20 μm (Merge) or 10 μm (Zoom).

We previously showed that Trio regulates the stability of endothelial cell-cell junctions [11]. To study the functionality of these serine residues in Trio on the stability of endothelial cell-cell junctions, we used thrombin to disrupt endothelial cell-cell junctions. Endothelial cells were transfected with the GFP-tagged WT and DD mutant and treated with thrombin for different time periods. Typically for these experiments, barrier function can be determined using electrical impedance sensing techniques. However, due to the large size of the Trio protein, we were unable to efficiently transfect or transduce the compete endothelial monolayer to reliably measure barrier function. Alternatively, immunofluorescent images of single transfected cells were recorded and analysed on morphological changes. TrioFL-WT expressing endothelial cells showed similar behaviour as non-transfected cells; namely, an increase in the number of F-actin stress fibres was detected after five minutes of thrombin stimulation (Figure 4A and S1B). After 30 minutes, the appearance of so-called focal adherens junctions (FAJ) is observed, resulting in loss of endothelial cell-cell contacts and intracellular gaps in the monolayer, followed by recovery of endothelial cell-cell junctions and monolayer reformation after 60 minutes of thrombin (Figure 4A and S1B). Interestingly, after 30 minutes, the cells expressing TrioFL-S1785D/S1786D still showed intact cell-cell junctions, whereas the surrounding non-transfected cells showed increased formation of FAJ and loss of cell-cell contacts. After 60 minutes, a recovery of the monolayer was observed (Figure 4B and S1C).

**Figure 5.**
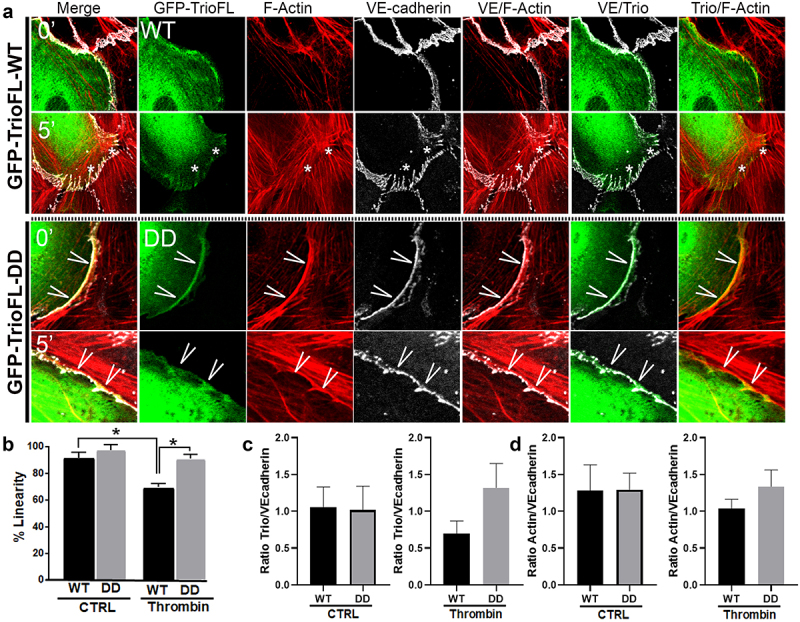
Phosphomimetic TrioFL-DD mutant co-localizes with VE-cadherin. (**a**) HUVECs were transfected with GFP-tagged TrioFL-WT or TrioFL-DD (phosphomimetic) mutant as indicated. Cells were treated with thrombin (1 U/mL) for five minutes and then fixed, permeabilized and stained for VE-cadherin (white) and F-actin in red. (**b**) Quantification of junctional linearity in TrioFL-WT and Trio-DD expressing cells before and after thrombin treatment. Experiment is carried out three times. Data are mean ± SEM. *p < 0.05. (**c**) the ratio between F-actin and GFP-TrioFL-WT or the DD mutant was quantified upon control (CTRL) or after 5 minutes of thrombin treatment. At least 10 cells expressing Trio constructs were analysed independently from each other. (**d**) the ratio between VE-cadherin and GFP-TrioFL-WT or the DD mutant was quantified upon control (CTRL) or after 5 minutes of thrombin treatment. At least 10 cells expressing Trio constructs were analysed independently from each other.

A more in-depth inspection revealed that the phosphomimetic mutant prevented loss of VE-cadherin-based cell-cell junctions, compared to TrioFL-WT by promoting the linear junctional phenotype (Figure 5A). Linearity was quantified and showed a significant increase in Trio-DD cells upon 5 minutes of thrombin treatment (Figure 5B). Moreover, we quantified the amount of fluorescent overlap between VE-cadherin and F-actin at control and 5-minute thrombin stimulation. These data showed that upon thrombin treatment, more F-actin co-localized with VE-cadherin in the cells that were transfected with the mutant compared to the WT-transfected endothelial cells. However, at control conditions there was no difference between the F-actin/VE-cadherin colocalization between the Trio constructs (Figure 5C). We furthermore analysed the amount of localization of Trio to VE-cadherin upon thrombin treatment. Here we found that Trio-DD co-localized stronger to VE-cadherin upon thrombin treatment than Trio-WT did (Figure 5D). These data indicate that Trio-S1785D/S1786D mutant attempts to prevent loss of endothelial cell-cell contacts upon thrombin treatment. In summary, we conclude that phosphorylation of the serine residues S1785/S1786 in RhoGEF Trio plays a dominant role in stabilizing endothelial cell-cell junctions.

## Discussion

In this study, we found two serine residues (S1785/S1786) in the RhoGEF Trio that controlled downstream thrombin signalling. We found that the thrombin-induced focal adherence junction (FAJ) phenotype in endothelial cells was largely blocked in cells that expressed the phosphomimetic Trio mutant, indicating that the phosphorylation status of Trio may stabilize endothelial cell-cell junctions.

Phosphorylation is involved in controlling the activity of many GEFs. The family of Vav GEFs is known to activate a variety of GTPases, including RhoA and Rac1, and is activated in response to tyrosine phosphorylation. In the non-phosphorylated, inactive (‘closed’) conformation of Vav, the C-terminus of Vav occludes the GTPase binding site and limits the catalytic output of Vav [15]. Tyrosine phosphorylation of Vav induces changes in the intramolecular architecture that opens the molecule into an active conformation [16–19].

Thus far, only the crystal structure of the isolated first enzymatic exchange domain of Trio, TrioGEF1, has been resolved [20,21] and it is still unclear how the full Trio protein is exactly folded. One potential mechanism for Trio activation may be the relief of an auto-inhibitory intramolecular interaction, like the regulation of Vav-GEFs by tyrosine phosphorylation. Elegant data from DeGeer and colleagues showed that blocking tyrosine phosphorylation of specific residues within the C-terminus of Trio prevented netrin-induced Rac1 activation in N1E–115 neuroblastoma cells [10]. We here show that the full C-terminal part of Trio, including the second GEF domain is not required for nucleotide exchange on Rac1 or RhoG in endothelial cells, suggesting that the intramolecular interaction between the *N*- and C-terminal part of Trio is not the mechanism of Rac1 activation upon thrombin stimulation. Interestingly and in line with our findings, enhanced tyrosine phosphorylation by inhibition of the tyrosine phosphatases by pervanadate treatment in cells that expressed Trio truncation mutants did not raise Rac1-GTP levels. One explanation is that the GTP loading on Rac1 was already maximal and therefore additional activation may not be possible. Similarly, expressing both TrioN and the GEF1 domain did not achieve any additional GTP loading on Rac1, indicating maximum loading. However, for the Trio full length construct, the GTP loading on Rac1 was not limiting. Yet also under these conditions no increase in Rac1-GTP loading was detected after pervanadate treatment. As our data showed no inhibition of Rac1 activation by expressing TrioC and TrioN together, we concluded from these data that Trio-GEF1 activity is not directly regulated through the relief of an auto-inhibitory intramolecular interaction to the GEF1 domain by the C-terminus of Trio or through general tyrosine phosphorylation.

Only a few studies have thus far linked Trio to serine phosphorylation. Debant and colleagues demonstrated Trio phosphorylation, mainly on serine residues, after phorbol ester or pervanadate treatment [9]. Xin and co-workers showed that Trio could potentially be phosphorylated by the serine kinase Cdk5, which was required for Rac1 activation [22]. Here, we show that stimulation of endothelial cells with thrombin leads to Trio phosphorylation on several serine residues, most prominently serines 1785 and 1786, located in between the two GEF domains. Thrombin is an inflammatory mediator that induces a rapid loss of cell-cell junctions and a drop in basal resistance of endothelial cell monolayers. This is followed by a Rac1-dependent recovery phase, wherein endothelial cell junctions are reformed and barrier function is restored. Interestingly, we observed that expression of a phosphomimetic Trio mutant prevented thrombin-induced breakdown of cellular junctions, suggesting that these serines are involved in Trio-mediated stabilization of endothelial junctions. Indeed, endothelial cells expressing the double mutants showed increased junction linearity upon thrombin treatment, indicative for increased junction stability [23]. The phosphorylation of serines 1785 and 1786 May therefore be important for minimizing the harmful effects of thrombin on endothelial cell junctions by concentrating Rac1 activity at cell-cell junctions. These findings are in line with previous studies from our lab that indicate that the reformation of junctions after thrombin stimulation was delayed in cells that were depleted for Trio [11]. Our novel data suggest that the rapid phosphorylation of Trio in response to thrombin and the consequent activation of Rac1 is essential to preserve stable linear junctions and potentially prime endothelial cells to repair cellular junctions more rapidly. Although it is still not known which kinase is responsible for thrombin-mediated serine phosphorylation of Trio, it is clear that specific Trio phosphorylation events can control local Rac1 activation.

## Materials and methods

### Antibodies

Monoclonal antibodies to Rac1 was obtained from BD Transduction Laboratories (Amsterdam, The Netherlands). Monoclonal antibodies to VE-cadherin (clone F8) and RhoA were purchased from Santa Cruz Biotechnology (Heidelberg, Germany). Monoclonal antibody to actin (clone AC-40) was purchased from Sigma (Zwijndrecht, The Netherlands). RhoG monoclonal antibody was a kind gift from Dr. J. Meller and Dr. M.A. Schwartz (University of Virginia, Charlottesville, USA). Monoclonal antibody for GFP (JL-8), secondary goat anti-rabbit IR 680, goat anti-mouse IR 800 and donkey anti-goat IR 800 antibodies were purchased from Licor Westburg (Leusden, The Netherlands). Secondary Alexa-labelled antibodies and Alexa-633-conjugated phalloidin and phalloidin-texas red were from Invitrogen (Breda, The Netherlands). Secondary HRP-conjugated goat anti-mouse, swine anti-rabbit and rabbit anti-goat antibodies were purchased from Dako (Heverlee, Belgium).

### Cell cultures, treatments and transfections

Human umbilical vein endothelial cells (HUVECs), purchased from Lonza, were cultured on fibronectin-coated dishes in EGM-2 medium, and supplemented with Singlequots (Lonza, Verviers, Belgium). HUVECs were cultured up to passage 9. HEK-293T cells were maintained in IMDM (Iscove’s Modified Dulbecco’s Medium) (BioWhittaker, Verviers, Belgium) containing 10% (v/v) heat-inactivated foetal calf serum (Invitrogen, Breda, The Netherlands), 300 mg/mL L-glutamine, 100 U/mL penicillin and streptomycin. Cells were cultured at 37°C and 5% CO_2_. HUVEC were treated with 1 U/mL thrombin (Sigma-Aldrich, St. Louis, USA) as indicated. Cells were transfected with expression vectors according to the manufacturer’s protocol with Trans IT-LT1 reagent (Myrus, Madison, WI, USA) or via electroporation (1 pulse, 1350 V, 30 msec) according to manufacturer’s protocol (Invitrogen).

### Confocal laser scanning microscopy

Cells were cultured on FN-coated glass coverslips and transfected or stimulated with 1 U/mL thrombin as indicated. After treatment, cells were washed with cold PBS, containing 1 mM CaCl_2_ and 0.5 mM MgCl_2_, and fixed with 4% (v/v) formaldehyde for 10 minutes. After fixation, cells were permeabilized in PBS supplemented with 0.2% (v/v) Triton X-100 for 10 minutes followed by a blocking step in PBS supplemented with 2% (w/v) BSA. Cells were incubated with primary and secondary antibodies and after each step washed with PBS. Fluorescent imaging was performed with a confocal laser-scanning microscope (LSM510/Meta; Carl Zeiss MicroImaging) using a 63× NA 1.40 or a 40× NA 1.30 oil lens.

### GTPase activity assays

Cells were lysed in 50 mM Tris, pH 7.4, 0.5 mM MgCl_2_, 500 mM NaCl, 1% (v/v) Triton X-100, 0.5% (w/v) deoxycholic acid (DOC), and 0.1% (w/v) SDS supplemented with protease inhibitors. Subsequently, lysates were cleared at 10.000 rpm for 10 minutes. GTP-bound Rac1 was isolated by rotating supernatants for 30 minutes with 30 μg of a biotinylated PAK1-CRIB peptide (corresponding to the Cdc42 and Rac1 interacting binding (CRIB) domain of PAK), coupled to streptavidin agarose (Price et al., 2003). GTP-bound RhoG was isolated by rotating supernatants for 30 minutes with 60 μg of a GST fusion protein containing the full-length RhoG effector ELMO (GST-ELMO), which was pre-coupled to glutathione sepharose beads (GE Healthcare, Zeist, The Netherlands) (van Buul et al., 2007; Wittchen and Burridge, 2008). Beads were washed five times in 50 mM Tris, pH 7.4, 0.5 mM MgCl2, 150 mM NaCl, 1% (v/v) Triton X-100 and boiled in SDS-sample buffer containing 4% β-mercapto-ethanol. Samples were analysed by SDS-PAGE. RhoA activation was measured using the RhoA effector Rhotekin (GST-Rhotekin), which was pre-coupled to glutathione sepharose beads and treated as described RhoG pulldown. For the precipitation of Trio, GST-Rac1-G15A fusion proteins are used, coupled to glutathione beads and treated as described for the RhoG pulldown.

### Fusion proteins

The fusion proteins GST-ELMO, GST-Rhotekin and GST-Rac1 G15A were purified from BL21 Escherichia coli cells (Agilent Technologies, Amstelveen, Netherlands) with glutathione – Sepharose 4B, as previously described (Ellerbroek et al., 2004). GST-fusion proteins were stored in 30% (v/v) glycerol at − 80°C.

### Junction linearity

To measure junction linearity, the length between two points was divided by the actual length between those same two points. Points were set as end points of junction regions, i.e. typical tri-cellular junction corners, and the junction was determined by VE-cadherin staining.

### Statistical analysis

Statistical comparisons between experimental groups were performed by the student t-test. A two-tailed p-value of ≤ 0.05 was considered significant. Unless otherwise stated, a representative experiment out of at least three independent experiments is shown.

## Supplementary Material

Supplemental Material

## References

[1] Burridge K, Wennerberg K. Rho and rac take center stage. Cell. 2004;116(2):167–179. doi: 10.1016/S0092-8674(04)00003-0 14744429

[2] Bos JL, Rehmann H, Wittinghofer A. Gefs and GAPs: critical elements in the control of small G proteins. Cell. 2007;129(5):865–877. doi: 10.1016/j.cell.2007.05.018 17540168

[3] Rossman KL, Der CJ, Sondek J. GEF means go: turning on RHO GTPases with guanine nucleotide-exchange factors. Nat Rev Mol Cell Biol. 2005;6(2):167–180. doi: 10.1038/nrm1587 15688002

[4] Wennerberg K, Der CJ. Rho-family GTPases: it’s not only Rac and Rho (and I like it). J Cell Sci. 2004;117(8):1301–1312. doi: 10.1242/jcs.01118 15020670

[5] Bar-Sagi D, Rotin D, Batzer A, et al. SH3 domains direct cellular localization of signaling molecules. Cell. 1993;74(1):83–91. doi: 10.1016/0092-8674(93)90296-3 8334708

[6] van Rijssel J, Kroon J, Hoogenboezem M, et al. The Rho-guanine nucleotide exchange factor Trio controls leukocyte transendothelial migration by promoting docking structure formation. Mol Biol Cell. 2012;23(15):2831–2844. 22696684 10.1091/mbc.E11-11-0907 PMC3408411

[7] van Rijssel J, Hoogenboezem M, Wester L, et al. The N-terminal DH-PH domain of Trio induces cell spreading and migration by regulating lamellipodia dynamics in a Rac1-dependent fashion. PLoS One. 2012;7:e29912. 22238672 10.1371/journal.pone.0029912 PMC3253119

[8] van Rijssel J, van Buul JD. Cell Adh Migr. 2012 Nov–Dec;6(6):482–487. 23076143 10.4161/cam.21418 PMC3547891

[9] Debant A, Serra-Pages C, Seipel K, et al. The multidomain protein Trio binds the LAR transmembrane tyrosine phosphatase, contains a protein kinase domain, and has separate rac-specific and rho-specific guanine nucleotide exchange factor domains. Proc Natl Acad Sci USA. 1996;93(11):5466–5471. doi: 10.1073/pnas.93.11.5466 8643598 PMC39269

[10] DeGeer J, Boudeau J, Schmidt S, et al. Tyrosine phosphorylation of the rho guanine nucleotide exchange factor trio regulates netrin-1/DCC-Mediated cortical axon outgrowth. Mol Cell Biol. 2013;33(4):739–751. doi: 10.1128/MCB.01264-12 23230270 PMC3571336

[11] Timmerman I, Heemskerk N, Kroon J, et al. A local VE-cadherin and Trio-based signaling complex stabilizes endothelial junctions through Rac1. J Cell Sci. 2015;128(16):3041–3054. 26116572 10.1242/jcs.168674

[12] van den Biggelaar M, Hernandez-Fernaud JR, van den Eshof BL, et al. Quantitative phosphoproteomics unveils temporal dynamics of thrombin signaling in human endothelial cells. Blood. 2014;123(12):e22–e36. doi: 10.1182/blood-2013-12-546036 24501219 PMC3962174

[13] Feig LA. Tools of the trade: use of dominant-inhibitory mutants of Ras-family GTPases. Nat Cell Biol. 1999;1(2):E25–E27. doi: 10.1038/10018 10559887

[14] Garcia-Mata R, Wennerberg K, Arthur WT, et al. Analysis of activated GAPs and GEFs in cell lysates. Methods Enzymol. 2006;406:425–437. 16472675 10.1016/S0076-6879(06)06031-9

[15] Bustelo XR. Regulation of Vav proteins by intramolecular events. Front Biosci. 2002;7(4):d24–d30. doi: 10.2741/A766 11779690

[16] Aghazadeh B, Lowry WE, Huang XY, et al. Structural basis for relief of autoinhibition of the dbl homology domain of proto-oncogene vav by tyrosine phosphorylation. Cell. 2000;102(5):625–633. doi: 10.1016/S0092-8674(00)00085-4 11007481

[17] Barreira M, Fabbiano S, Couceiro JR, et al. The C-Terminal SH3 domain contributes to the intramolecular inhibition of Vav family proteins. Sci. 2014;7(321):ra35. doi: 10.1126/scisignal.2004993 24736456

[18] Lopez-Lago M, Lee H, Cruz C, et al. Tyrosine Phosphorylation Mediates Both Activation and Downmodulation of the Biological Activity of Vav. Mol Cell Biol. 2000;20(5):1678–1691. doi: 10.1128/MCB.20.5.1678-1691.2000 10669745 PMC85351

[19] Yu B, Martins IR, Li P, et al. Structural and energetic mechanisms of cooperative autoinhibition and activation of Vav1. Cell. 2010;140(2):246–256. doi: 10.1016/j.cell.2009.12.033 20141838 PMC2825156

[20] Chhatriwala MK, Betts L, Worthylake DK, et al. The DH and PH domains of trio coordinately engage Rho GTPases for their efficient activation. J Mol Biol. 2007;368(5):1307–1320. doi: 10.1016/j.jmb.2007.02.060 17391702 PMC1890047

[21] Skowronek KR, Guo F, Zheng Y, et al. The C-terminal basic tail of RhoG assists the guanine nucleotide exchange factor trio in binding to phospholipids. J Biol Chem. 2004;279(36):37895–37907. doi: 10.1074/jbc.M312677200 15199069

[22] Xin X, Ferraro F, Back N, et al. Cdk5 and Trio modulate endocrine cell exocytosis. J Cell Sci. 2004;117(20):4739–4748. doi: 10.1242/jcs.01333 15331630

[23] Ando K, Fukuhara S, Moriya T, et al. Rap1 potentiates endothelial cell junctions by spatially controlling myosin II activity and actin organization. J Cell Bio. 2013;202(6):901–916. doi: 10.1083/jcb.201301115 24019534 PMC3776352

