# Supplementary material for: Serine phosphorylation of the RhoGEF Trio stabilizes endothelial cell-cell junctions
Source: Small GTPases. 2023 Aug 1;14(1):45–54. doi: 10.1080/21541248.2023.2242166 (PMC10399470; doi:10.1080/21541248.2023.2242166)
Supplement: Supplemental Material [file KSGT_A_2242166_SM8343.docx]

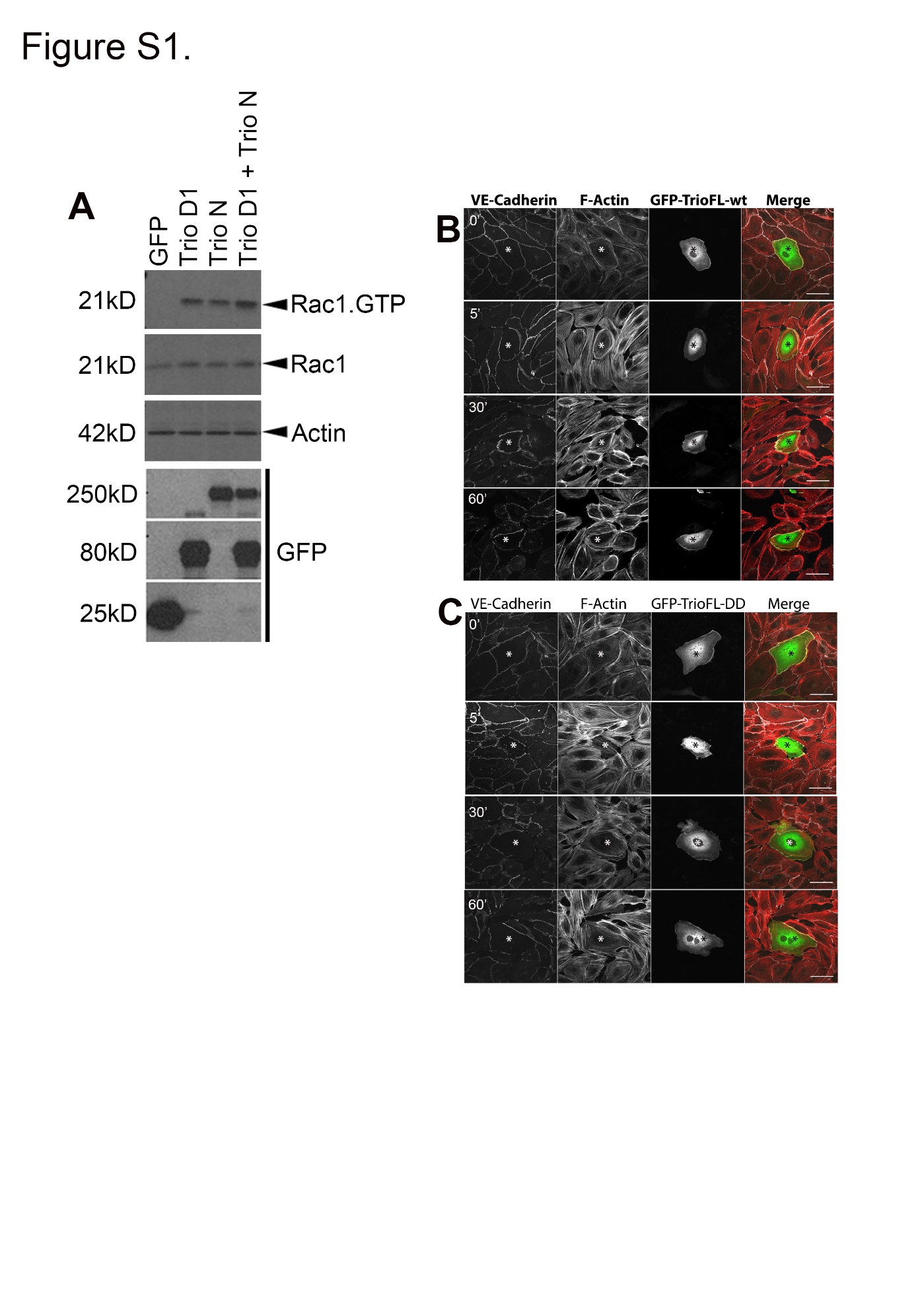


**Figure S1. Trio activates endogenous Rac1. (A)** Hek293-T cells were transfected as indicated and Rac1 activity was measured using CRIB as a bait, showing that expression of TrioD1 and TrioN together did not further increase Rac1.GTP levels, indicating that Rac1 is the limiting factor. Upper panel shows pull down results, lower panels show expression of proteins as indicate din total cell lysates. (**B**) **Trio-phosphomimetic (DD) mutant prevents thrombin-induced cortical F-actin bundles.** HUVECs were treated for 5 minutes with thrombin, fixed and stained for F-actin (red) and in green GFP-tagged Trio as indicated. Reduced cortical F-actin bundles are detected in the cells that express Trio-DD, compared to cells that express wt. (**C**) HUVECs were treated with thrombin for the period indicated in upper left corner, fixed and stained for F-actin (red), VE-cadherin in white and GFP-tagged Trio as indicated in green. Reduced loss of cell-cell junctions was detected in the cells that express Trio-DD (**C**), compared to cells that express wt.
